# Effectiveness of manual therapy for cervical radiculopathy, a review

**DOI:** 10.1186/s12998-016-0126-7

**Published:** 2016-12-09

**Authors:** E. J. Thoomes

**Affiliations:** Fysio-Experts Physical Therapy Clinic, Hazerswoude, The Netherlands

## Abstract

Manual therapy is often used for patients with neck pain with or without radicular symptoms. There is sparse evidence on the effectiveness in cervical radiculopathy. The aim of this study was to assess current levels of evidence on the effectiveness of manual therapy interventions for patients with cervical radiculopathy.

Electronic data bases were systematically searched for clinical guidelines, reviews and randomised clinical trials (RCTs) reporting on the effectiveness of manual therapy for patients with cervical radiculopathy. Eight relevant reviews, two guidelines and two recent RCTs, that had not yet been included in either, were retrieved. The overall quality of the evidence of included studies was evaluated using the GRADE method. Most interventions were only studied in one single RCT.

There is low level evidence that cervical manipulation and mobilisation as unimodal interventions are effective on pain and range of motion at the immediate follow up, but no evidence on the effectiveness of thoracic manipulation or mobilisation as unimodal interventions. There is low level evidence that a combination of spinal mobilisation and motor control exercises is more effective on pain and activity limitations than separate interventions or a wait-and-see policy. There is low level evidence of the effectiveness of cervical mobilisation with a neurodynamical intent as unimodal intervention, on the effectiveness of a multimodal intervention with neurodynamic intent on pain activity limitations and global perceived effect compared to a wait-and-see policy. There is also low level evidence that a multimodal intervention consisting of spinal and neurodynamic mobilisations and specific exercises is effective on pain in patients with CR. There is low level evidence that traction is no more effective than placebo traction.

## Background

Cervical radiculopathy (CR) is a term used to describe radiating pain in the arm with motor, reflex and/or sensory changes (such as paraesthesiae or numbness), provoked by neck posture(s) and/or movement(s) [1, 2]. It is most commonly caused by a cervical disc herniation or spondylotic changes such as bone spurs, resulting in nerve root compression and /or inflammation [1, 3].

There is sparse epidemiological data on the incidence and prevalence of CR. An annual age-adjusted incidence rate of 83.2 per 100,000 persons (107.3 for men and 63.5 for women) with a peak incidence in the 5^th^ and 6^th^ decade in both genders has been reported [4].

Little is known about the natural course of CR. A recent systematic review reported that patients with CR due to a cervical disc herniation substantially improved on levels of pain and activity within the first 4 to 6 months and were able to return to their normal activities after 24 to 36 months [5].

As surgery is associated with a small but definite risk [6], conservative management is a suggested first treatment choice in the absence of serious neurological deficits [7, 8].

Manual therapy is form of conservative treatment provided by specialized physical therapists, chiropractors, osteopaths and sometimes by other health care providers. It is thought to produce a variety of effects including improved tissue extensibility and range of motion; relaxation; altered muscle function; modulation of pain; and reduction of soft tissue swelling and inflammation [9, 10].

Research on the effectiveness of manual therapy treatment of CR is also sparse. Although some authors added either manual therapy as an intervention or the disorder CR disorder as a subgroup in their review, only two [11, 12] looked at manual therapy in general for patients with CR alone.

The aim of the current study was to assess the effectiveness of manual therapy for patients with CR compared to placebo, no treatment, other forms of conservative care or surgery on patient outcome such as pain, disability, return to work, global perceived effect or quality of life.

## Methods

### Selection criteria

**Fig. 1:**
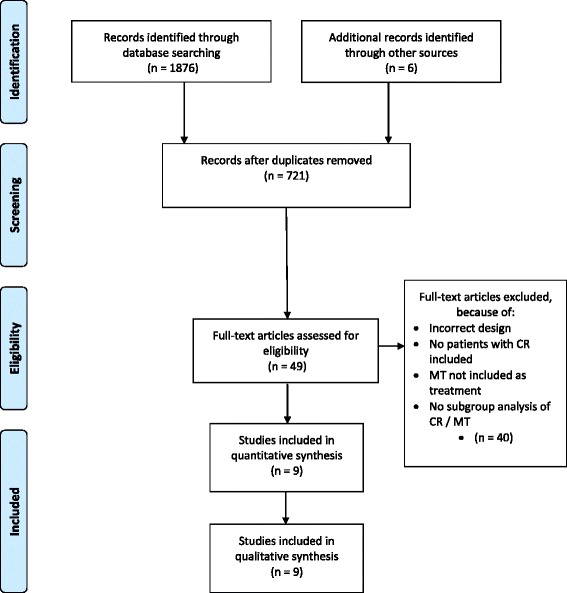
PRISMA flow chart of included studies

The PRISMA guidelines for reporting were used [13]. The studied population should consist of, or separately report on, patients with CR who had at least partially received manual therapy as an intervention. The manual therapy intervention should be compared to placebo, no treatment, other forms of conservative care or surgery on patient outcomes. Studies were included that used at least one of the primary outcome measures that were considered to be the most important, namely: pain intensity, global perceived effect (e.g. proportion of patients recovered, subjective improvement of symptoms), disability (e.g. Neck Disability Index, Bournemouth Neck Questionnaire), return to work (e.g. days off work) or quality of life. Outcomes of physical examinations (e.g. range of motion, spinal flexibility, muscle strength, upper limb nerve tension testing), and psycho-social outcomes (e.g. anxiety, depression, pain behaviour) were considered as secondary outcomes. Randomised clinical trials (RCTs), (systematic) reviews or published clinical guidelines were considered eligible. Abstracts for which full reports were not available were excluded (See Fig. 1).

### Search strategy

A research librarian together with the review author performed the electronic search that followed the recommendations in the Cochrane Handbook for Systematic Review of Interventions [14]. Electronic searches included PubMed, the Cochrane Library, Embase, Cinahl, PEDro, en de National Guideline Clearinghouse from inception to November 2015. We used MeSH (Medline), Thesaurus (EMBASE, CINAHL) and free text words. Combinations were made based on a) localisation (cervical); b) disorder (radiculopathy) and c) intervention (conservative treatment, non-surgical, non-invasive, manual therapy, physiotherapy, physical therapy, exercise, rest, traction, mobilisation/ mobilization, manipulation, chiropractic). Manual searches of review bibliographies and reference lists of primary studies were undertaken to search for possible studies not captured by the electronic searches. Titles and abstracts were screened for eligibility. Next, full text papers were assessed to ascertain whether the study met the inclusion criteria regarding design, participants, and interventions.

### Quality assessment

In an effort to minimize bias from having only one rater, the PEDro database and scoring system was used to assess the quality of the individual studies. The review author assessed scores for the studies for which no PEDro score was available.

### Data extraction

Data with respect to participants, in- & exclusion criteria, interventions, outcome measures and results of the included RCTs were extracted.

### Strength of the evidence

The overall quality of the evidence was evaluated using the GRADE method. The quality of the evidence was based upon five principal factors: 1) limitations in study design (downgraded when >25 % of the participants were from studies with a low methodological quality according to the PEDro scale), 2) inconsistency of results [downgraded when there was statistical heterogeneity (I^2^ > 40 %) or inconsistent findings (defined as ≤75 % of the participants reporting findings in the same direction)], 3) indirectness (e.g. generalizability of the findings), 4) imprecision (downgraded when the total number of participants across studies were <300 for each outcome) and 5) other considerations, such as reporting bias. The quality of the evidence was downgraded by one level when one of the factors described above was met [15]. Single studies were considered inconsistent and imprecise (i.e. sparse data) and providing “low quality evidence”, which could be further downgraded to “very low quality evidence” if there were also limitations in design or indirectness. The following grading of quality of the evidence was applied:

- High quality: further research is very unlikely to change confidence in the estimate of effect.
- Moderate quality: further research is likely to have an important impact on confidence in the estimate of effect and may change the estimate.
- Low quality: further research is very likely to have an important impact on confidence in the estimate of effect and is likely to change the estimate.
- Very low quality: there is much uncertainty about the estimate.
- No evidence: no evidence from any RCTs.

This structured approach was intended to minimize the potential bias of having only one rater.

## Results and Discussion

### Study selection

Eight relevant (systematic) reviews were retrieved, two guidelines on the effectiveness of manual therapy (either as a subgroup or as part of a population of non-specific neck pain) and two recent RCTs that had not yet been included in either (see Fig. 1).

#### Evidence from (systematic) reviews

Two reviews specifically looked at the manual therapy treatment of patients with CR (see Table 1) [11, 12].

**Table 1:** Systematic reviews on manual therapy in patients with CR

Author, year; included studies	Review conclusion
Boyles, [11];	
Cleland [16]; Persson [17]; Ragonese [18]; Young [19]	Using manual therapy techniques in conjunction with therapeutic exercise is effective in regard to increasing function, as well as AROM, while decreasing levels of pain and disability.
Rodine, [12];	
BenEliyahu [22]; Howe [20]; Murphy [20]	Currently, randomized trials in the field of CR are lacking. Despite this, existing literature does provide support for the cautious application of (chiropractic) HVLA procedures in cases of confirmed or suspected CR.

*AROM* Active Range of Motion, *HVLA* High Velocity Low Amplitude

One review included 4 studies [16–19] in which combinations of different techniques like thrust and non-thrust mobilisations, neurodynamic techniques and muscle energy techniques were used. The authors concluded that manual therapy techniques combined with specific exercises were effective in improving function, active range of motion and in reducing pain and restrictions in activity and limitations in participation [11].

Another review on chiropractic High Velocity Low Amplitude (HVLA) manipulations, reflecting on chiropractic treatment practices, included 1 RCT [20], a prospective cohort study [21] and a case series [22]. They reported finding hardly any evidence of HVLA in patients with CR [12].

Six other reviews [23–28] assessed the effectiveness of manual therapy as a form of conservative treatment for patients with neck pain and also included patients with CR, but not as a separate subgroup (see Table 2). One of these reviews compared the effectiveness of spinal mobilisations and/or manipulations with other conservative treatments in patients with cervical or lumbar radiculopathies [27]. The authors included 5 studies [20, 29–32] and concluded there was very low level evidence that manipulation/mobilisation was no more effective than other conservative therapies. Another review concluded that in both patients with or without CR, the long-term effectiveness of manual therapy combined with specific exercises on the level of pain an global perceived effect was better than no treatment [28]. Other reviews also concluded there either was insufficient evidence or there was low level evidence that manipulation/mobilisation was no more effective than other conservative therapies for patients with CR [23–26, 28].

**Table 2:** Systematic reviews on the manual therapy treatment of patients with neck pain, also including patients with CR

Author, year; studies including patients with CR	Review conclusion
Guzman, [25] & Hurwitz, [78];	
Brodin [38] Hoving [79]; Persson [17, 80]	There is insufficient evidence to support a decision for or against the use of a specific conservative treatment (including manual therapies) in patients with CR
D’Sylva [23]	
Brodin [38]; Hurwitz 2002; Kogstad [81];	There is low level evidence of difference in pain relief, functional improvements or global perceived effect when the combination of manual therapy and physical medicine modalities is compared to placebo, exercise, various combinations of manipulation, education and rare collar use, or physiotherapy applications in patients with or without CR.
Gross [24]	
Howe1985; Hurwitz 2002	No separate conclusion that cervical and /or thoracic manipulation is more effective for patients with CR.
Miller, [28];	
Allison [30]; Brodin [38]; Hoving [79]; Persson [17, 80]	Manipulation or mobilization and exercise produces a greater long-term improvement in pain and global perceived effect when compared to no treatment for chronic neck pain, subacute/ chronic neck pain with cervicogenic headache, and chronic neck pain with or without radicular findings. There was insufficient evidence available to draw any conclusions for neck disorder with radicular findings.
Leiniger, 27];	
Allison [30]; Howe 1985; Moretti [32]; Shin [29]; Walker [31]	The evidence for manipulation or mobilization for cervical spine–related extremity symptoms is very low in quality for all included comparison therapies. Thus, conclusions regarding effectiveness cannot be made

Two systematic reviews evaluated the effectiveness of conservative therapies specifically for patients with CR and included manual therapy as an intervention (see Table 3) [33, 34]. One found very low level evidence than manual therapy combined with exercises was more effective at short term follow up (3 weeks) than either manual therapy or exercises alone on level of pain and activity limitations [33]. Another review (including 11 RCTs, two of which were of low risk of bias), concluded that, based on very low to low level evidence, no single intervention seemed to be superior or consistently more effective than others [34]. Manual therapy was assessed in two [18, 19] of the included RCTs. One study assessed the effectiveness of surgical interventions, comparing them to conservative management, but also evaluated the individual conservative treatments [35]. The authors concluded that the literature yielded no studies to adequately address the role of physical therapy / manual therapy or exercise in the management of cervical radiculopathy from degenerative disorders.

**Table 3:** Systematic reviews including manual therapy in the treatment of patients with CR

Author, year; included studies using manual therapies	Review conclusion
Salt, [33];	
Allison [30]; Coppieters [39]; Howe 1985; Walker [31]; Ragonese [18]	There is inconclusive evidence for the effectiveness of noninvasive management of cervicobrachial pain. Potential benefits were indicated in the provision of manual therapy and exercise approaches to reduce pain. Traction was no more effective than comparators in reducing pain. Effects of non-invasive management on function and disability were mixed.
Thoomes, [34];	
Persson [17]; Ragonese [18]; Young [19]	On the basis of low-level to very low-level evidence, no 1 conservative intervention seemed to be superior or consistently more effective than other interventions.

In a recent best evidence clinical guideline, the American Physical Therapy Association (APTA) concluded there is moderate evidence for the effectiveness of neurodynamic mobilisations and that, based on low-level evidence, thoracic manipulations and traction can be considered for patients with CR [36]. A multidisciplinary guideline “complaints of arm, neck and/or shoulder” (CANS) describes CR as a subgroup, but makes no statement concerning manual therapy for patients with CR [37].

#### Evidence from RCTs

All the above mentioned reviews included a total of 7 RCTs [18–20, 29, 30, 38, 39] that compared the effectiveness of manual therapy to other interventions specifically in patients with CR. One RCT with 2 studies [31, 40] evaluated the effectiveness of manual therapy in patients with neck pain with or without CR.

Two recent RCTs had not yet been included in a review [41, 42]. The authors of one RCT concluded that a combination of a cervical “lateral glide’ mobilisation technique [43, 44] and neurodynamic mobilisation was more effective at short-term follow up on pain and disability than a wait-and-see policy [42]. Results of one other RCT suggested that based on the size of the treatment effect on pain and activity limitations, a combination of manual therapy and specific exercises was more effective at short term follow up than a wait-and-see policy [41].

### Level of evidence

Five out of the nine included RCTs were of high methodological quality, as assessed using the PEDro scoring system (see Table 4).

**Table 4:** Methodological quality assessment of individual studies based on PEDro scores

	Random allocation	Concealed allocation	Baseline comparability	Blind subjects	Blind therapists	Blind assessors	Adequate follow-up	Intention-to-treat analysis	Between-group comparisons	Point estimates and variability	PEDro score	Methodological quality
Allison, [30]	Y	N	N	N	N	Y	N	Y	Y	Y	5/10	L
Brodin, [38]	Y	N	N	N	N	N	Y	N	Y	Y	4/10	L
Coppieters, [39]	Y	Y	Y	N	N	Y	N	Y	Y	Y	7/10	H
Howe, [20]	Y	Y	Y	N	N	Y	Y	N	Y	Y	7/10	H
Langevin, [41]	Y	Y	Y	N	N	N	Y	Y	Y	Y	7/10	H
Nee, [42]	Y	Y	Y	N	N	Y	Y	Y	Y	Y	8/10	H
Ragonese, [18]	Y	Y	N	N	N	Y	Y	N	Y	Y	6/10	L
Shin, [29]	Y	Y	N	N	N	N	Y	N	Y	N	4/10	L
Young, [19]	Y	Y	Y	N	N	Y	Y	Y	Y	Y	8/10	H

*Y* Yes, *N* No, *H* High, *L* Low

An overview of the study characteristics of these RCTs evaluating manual therapy specifically for patients with CR is presented in Table 5.

**Table 5:** RCTs including manual therapy in the treatment of patients with CR

Author, year; included patients	Intervention & Control	Study conclusion
Howe, [20]; *n =* 52	I: Manipulation and /or injection + NSAID (*n =* 26) C: NSAID (*n =* 26)	Manual therapy provided immediate significant pain reduction, but at the 1 week follow up there was no between-group difference anymore.
Brodin, [38]; *n =* 63	I 1: Mobilisation (*n =* 21) I 2: Electrotherapy en massage (*n =* 21) C: Wait & see (*n =* 21)	Segmental mobilisation was more effective than a placebo or a wait & see policy on the level of pain and range of motion.
Allison, [30]; *n =* 30	I 1: Thoracic & articular mobilisation (*n =* 10) I 2: Neurodynamic mobilisation (*n =* 10) C : Wait & see (*n =* 10)	Manual therapy combined with neurodynamic mobilisation is effective in improving pain intensity, pain quality scores and functional disability levels
Coppieters, [39]; *n =* 20	I: Cervical mobilisation with neurodynamic intent (*n =* 10) C: Therapeutic ultrasound (*n =* 10)	When a cervical dysfunction could be regarded as a cause of the neurogenic disorder or as a contributing factor that impedes natural recovery, a cervical lateral glide mobilisation has positive immediate effects in patients with subacute CR.
Shin, [29]; *n =* 26	I: Chuna Manual Therapy (CMT, *n =* 13) C: Cervical Traction (CT, *n =* 13)	Both CT and CMT reduce the level of pain, but CMT was found to be more effective than CT.
Ragonese, [18]; *n =* 30	I 1: manual therapy (*n =* 10) I 2: exercise (*n =* 10) I 3: combination (*n =* 10)	A combination of segmental spinal mobilisation and specific motor control exercises is more effective on pain and activity limitations than separate interventions of manual therapy or exercises alone.
Young, [19]; *n =* 81	I: Traction & manual therapy & exercise (*n =* 45) C: Placebo traction & manual therapy & exercise (*n =* 36)	At the 2 and 4 week follow up there was so significant difference between groups on pain and activity limitations. Note: manual therapy consisted of thoracic manipulation and thoracic & cervical mobilisation
Nee, [42]; *n =* 60	I: Cervical mobilisation with neurodynamic intent & peripheral neurodynamic mobilisations (*n =* 40) C : Wait & see (*n =* 20)	At the 2 week follow up the intervention group reported substantial reductions in neck pain, arm pain, and activity limitations.
Langevin, [41]; *n =* 36	I : Cervical mobilisation + specific exercises, both aimed at opening IVF (*n =* 18) C: General mobilisation and exercises NOT aimed at opening IVF (*n =* 18)	Preliminary results based on the size of the treatment effect, suggest that at the 4 & 8 week follow up, a combination of manual therapy and motor control exercises is more effective on pain and activity limitations than a wait-and-see policy. There is no difference between general mobilisation or mobilisation aimed at opening the IVF.

*I* Intervention, *C* Control, *ROM* range of motion, *ULNT* upper limb neural test, *IVF* intravertebral foramen, *GPE* Global Perceived Effect, *NDI* Neck Disability Index, *PSFS* Patient Specific Functional Scale

### Specific manual therapy interventions

#### Unimodal interventions

##### Cervical manipulation as a unimodal therapy

One RCT of high methodological quality compared the effectiveness of cervical manipulation to NSAIDs in patients with CR [20]. The study reported a significant decrease in pain directly following treatment, but no significant difference was retained at 1 and 3 week follow up. In conclusion, there is low level evidence from one study of high methodological quality that cervical manipulation as unimodal intervention is effective on pain immediately after treatment but not at longer term follow up (see Table 6).

**Table 6:** Overview of effectiveness of manual therapy treatments

Intervention	Effectiveness	Level of evidence
Unimodal		
Cervical manipulation as unimodal therapy	More effective at short term follow up (<1 week) on pain than NSAIDs	Low level evidence from 1 study of high methodological quality (Howe, [20])**.**
Thoracic manipulation as unimodal therapy	Unknown	No RCTs found
Cervical mobilisation as unimodal therapy	More effective at immediate follow up than a placebo or a wait&see policy on pain and range of motion.	Very low level evidence from 1 study of low methodological quality (Brodin, [38]).
*Thoracic mobilisations as* unimodal therapy	Unknown	No RCTs found
Cervical mobilisation with a neurodynamical intent as unimodal intervention	Immediate increase in elbow extension during an ULNT and a decrease in the area of symptom distribution, and pain intensity.	Low level evidence from 1 study of high methodological quality (Coppieters, [39])**.**
Multimodal		
Combined joint mobilisation and specific (motor control) exercises	More effective at short term follow up (<4 weeks) than either manual therapy or exercise alone or wait & see on pain and activity limitations	Low level evidence from 2 studies, 1 of high (Langevin, [41]) and 1 of low (Ragonese, [18]) methodological quality.
Multimodal intervention with neurodynamic intent	More effective at short term follow up (<4 weeks) than wait & see policy on pain and global perceived effect	Low level evidence from 1 study with of high methodological quality (Nee, [42]**)**
Multimodal intervention with combined (neurodynamic, joint, muscle) intent	More effective at short term (3 - 8 weeks) follow up on pain	Low level evidence from 2 studies of low methodological quality (Ragonese, [18]; Allison, [30]**)**
Cervical traction combined with manual therapy and exercises	At the short term follow up (<4 weeks) no significant difference between traction or placebo traction	Low level evidence from 1 study of high methodological quality (Young, [19])

##### Thoracic manipulation as a unimodal therapy

No studies were found evaluating the effect of thoracic manipulation as a unimodal therapy in patients with CR. In conclusion, there is no evidence on the effectiveness of thoracic manipulation as a unimodal intervention in patients with CR.

##### Cervical traction

One study of low methodological quality compared cervical traction to Chuna Manual Therapy, a traditional Korean form of manual therapy. They reported improvement in both groups at the 2 week follow up, slightly favouring the manual therapy group [29].

##### Cervical mobilisation as a unimodal intervention

One RCT of low methodological quality evaluated the effectiveness of cervical mobilisation as a unimodal intervention in patients with CR [38]. This study reported that segmental mobilisation was more effective at immediate follow up than a placebo or a wait-and-see policy on pain and range of motion (ROM). In conclusion, there is very low level evidence from one study of low methodological quality that cervical mobilisation as a unimodal intervention is more effective at immediate follow up than a placebo or a wait-and-see policy on pain and ROM in patients with CR.

##### Thoracic mobilisation as a unimodal intervention

No studies were found evaluating the effectiveness of thoracic mobilisation as a unimodal intervention in patients with CR. In conclusion, there is no evidence on the effectiveness of thoracic mobilisation as a unimodal intervention in patients with CR.

##### Cervical mobilisation with a neurodynamic intent as unimodal intervention

One study of high methodological quality compared the immediate effect of a cervical mobilisation with a neurodynamic intent (‘Elvey’ or lateral glide) as a unimodal intervention with ultrasonography in patients with CR. They reported an increase in elbow extension during an Upper Limb Neural Tension test (ULNT) and a decrease in the area of symptom distribution, and pain intensity directly after treatment [39]. No studies were found evaluating the effect of neurodynamic mobilisations by using the arm as unimodal intervention. In conclusion, there is low level evidence from one study of high methodological quality of the effectiveness of cervical mobilisation with a neurodynamic intent as unimodal intervention in patients with CR.

#### Multimodal interventions

Manual therapy in RCTs of patients with CR is often an umbrella term encompassing multimodal interventions such as cervical manipulations and mobilisations combined with thoracic manipulations/mobilisation, traction, massage, neurodynamic mobilisation and specific exercises [18, 19, 30, 31, 36, 40, 41].

##### Combined joint mobilisation and specific exercises

Results from one study of high methodological quality suggested that, based on the size of the treatment effect on pain and activity limitations, a combination of manual therapy and motor control exercises was more effective at short term follow up (4 and 8 weeks) than a wait-and-see policy [41]. One study of low methodological quality reported more effectiveness of a combination of segmental spinal mobilisation and specific motor control exercises on pain and activity limitations than separate interventions of manual therapy or exercises alone in patients with CR [18]. In conclusion, there is low level evidence from two studies, one of low and one of high methodological quality, that a combination of spinal mobilisation and motor control exercises is more effective on pain and activity limitations than separate interventions or a wait-and-see policy.

##### Cervical traction combined with manual therapy and exercises

One study of high methodological quality compared the effectiveness of traction or placebo traction added to a regime of cervical mobilisation, thoracic manipulation and exercises. At the 2 and 4 week follow up there were no significant differences on pain or activity limitations [19]. In conclusion, there is low level evidence from one study of high methodological quality that traction is no more effective than placebo traction.

##### Multimodal intervention with neurodynamic intent

One study of high methodological quality s compared the effectiveness of cervical mobilisations with a neurodynamic intent combined with neurodynamic mobilisations using the shoulder and arm, to a wait-and-see policy [42]. At the 4 week follow up the experimental group had improved more on pain, activity limitations and global perceived effect (GPE) than the control group. In conclusion, there is low level evidence from one study of high methodological quality on the effectiveness of a multimodal intervention with neurodynamic intent on pain, activity limitations and GPE compared to a wait-and-see policy.

##### Multimodal intervention with combined (neurodynamic, joint, muscle) intent

Two studies of low methodological quality compared the effectiveness of a multimodal intervention (cervical, thoracic, neurodynamic and/or muscular mobilisations and exercises) to other conservative interventions in patients with CR [18, 30]. One study with a cross-over design investigated direct and indirect forms of active manual therapy interventions combined with home exercises. Both interventions demonstrated significant improvements in pain and disability compared to a wait-and-see policy [30]. The other study compared a manual therapy approach to motor control exercises and a combination of both. They included neurodynamic mobilisations in the manual therapy and combined groups and reported the group receiving the combination of manual techniques and exercises demonstrating the greatest improvements [18].

In conclusion, there is low level evidence from two studies of low methodological quality that a multimodal intervention consisting of spinal and neurodynamic mobilisations and specific exercises is effective on pain in patients with CR.

## Discussion

This study aimed to assess the effectiveness of manual therapy interventions for patients with CR in comparison to other conservative treatments, placebo interventions or a wait-and-see policy. The overall level of evidence for any intervention is low. This is mainly due to the fact that most interventions have only been evaluated in one single study and some of these were of low quality, which seriously impedes the firm drawing of conclusions.

There is a paucity of evidence for individual interventions or for combinations of interventions. From the few studies that were conducted, it would seem that multimodal management strategies are generally more effective than unimodal interventions. Several reviews and guidelines also conclude that a multimodal management strategy, comprising of spinal and neurodynamic mobilisation and specific exercises is the more effective conservative treatment for patients with CR [2, 11, 25, 26, 33, 36, 45, 46].

The preference of a multimodal approach is in agreement with recent publications on the effectiveness of conservative treatments of a variety of musculoskeletal disorders [47–54]. It also does justice to the integration in contemporary physiotherapy practice of all aspects of health as are outlined in the International Classification of Functioning, Disability and Health (ICF) [55].

In general, conservative treatments are not aimed at the pathology itself (in case of CR degenerative spondylosis or disc herniation), but rather at the predictable ensuing consequences. As radiculopathy is a neurological state in which conduction is blocked along a spinal nerve or its roots, it is not defined by pain alone, but also by neurological signs which can consist of motor, reflex and/or sensory changes such as paraesthesiae or numbness [56, 57]. Motor changes in the form of wasting of key-muscles present a therapeutic long term goal.

Chronic pain is also associated with CR. Specific dysfunctions in local muscles of patients with chronic pain have been documented, resulting in a loss of local motor control and direction specificity [58–62]. Changes in mechanical and dynamic properties of peripheral nerves due to pain and/or inflammation, although still an area needing more research, have also been documented [63–68].

## Strengths and limitations

A limitation of this study is having only one rater, so that a ‘risk of bias’ assessment according to Cochrane Collaboration guidelines could not be executed. Instead, the PEDro scale of quality assessment was used. In defence, all trials on PEDro are independently assessed for quality and there is preliminary evidence of the convergent and construct validity of the PEDro total score and the construct validity of eight individual scale items [69]. Another limitation is a single rater assessing the level of evidence, but again the strict prescriptive system of the GRADE system suggests a fair level of confidence in the presented outcomes.

Only two studies on cervical traction were included in this review as they were the only ones including manual therapy as an intervention [19, 29]. One other study that included manual therapy was excluded as it was a case series and not a randomized trial [70]. Other studies have evaluated the effectiveness of cervical traction in treating CR, but none included a form of manual therapy [71–77]. A recent review that evaluated these studies, concluded there was low-level evidence that traction is no more effective than placebo traction and very low level-evidence that intermittent traction was no more effective than continuous traction, thereby questioning the effectiveness of traction for patients with CR in general [34].

## Conclusions

In patients with CR:

- there is low level evidence that cervical manipulation as unimodal intervention is effective on pain directly after treatment but not at longer term follow up,
- there is no evidence on the effectiveness of thoracic manipulation or mobilisation as a unimodal intervention,
- there is very low level evidence that cervical mobilisation as a unimodal intervention is more effective at immediate follow up than a placebo or a wait-and-see policy on pain and ROM,
- there is low level evidence of the effectiveness of cervical mobilisation with a neurodynamic intent as unimodal intervention,
- there is low level evidence that a combination of spinal mobilisation and motor control exercises is more effective on pain and activity limitations than separate interventions or a wait-and-see policy,
- there is low level evidence that traction is no more effective than placebo traction,
- there is low level evidence on the effectiveness of a multimodal intervention with neurodynamic intent on pain activity limitations and GPE compared to a wait-and-see policy,
- there is low level evidence that a multimodal intervention consisting of spinal and neurodynamic mobilisations and specific exercises is effective on pain in patients with CR.

There is a lack of evidence for the effectiveness of manual therapy in patients with CR. Nearly all interventions have only been studied once and even then some in a low quality study only. Just two manual therapy interventions have been studied twice and then as part of multimodal interventions. Clearly there is a need for repeated high quality studies to be able to give patients and health care providers evidence based advice on management choices.

## Abbreviations

APTA: American Physical Therapy Association
CANS: Complaints of Arm, Neck or Shoulder
CR: Cervical Radiculopathy
GPE: Global Perceived Effect
GRADE: Grading of Recommendations Assessment, Development and Evaluation
ICF: International Classification of Function
NSAIDs: Non Steroid Anti Inflammatory Drugs
PEDro: Physiotherapy Evidence Database
RCT: Randomized Clinical Trial
ROM: Range of Motion
